# Learning-Dependent Dendritic Spine Plasticity Is Reduced in the Aged Mouse Cortex

**DOI:** 10.3389/fncir.2020.581435

**Published:** 2020-11-26

**Authors:** Lianyan Huang, Hang Zhou, Kai Chen, Xiao Chen, Guang Yang

**Affiliations:** ^1^Department of Anesthesiology, New York University School of Medicine, New York, NY, United States; ^2^Guangdong Provincial Key Laboratory of Brain Function and Disease, Zhongshan School of Medicine, Sun Yat-Sen University, Guangzhou, China; ^3^Department of Anesthesiology, Columbia University Medical Center, New York, NY, United States

**Keywords:** aging, dendritic spine, synaptic plasticity, learning, two-photon imaging, cortex

## Abstract

Aging is accompanied by a progressive decrease in learning and memory function. Synaptic loss, one of the hallmarks of normal aging, likely plays an important role in age-related cognitive decline. But little is known about the impact of advanced age on synaptic plasticity and neuronal function *in vivo*. In this study, we examined the structural dynamics of postsynaptic dendritic spines as well as calcium activity of layer 5 pyramidal neurons in the cerebral cortex of young and old mice. Using transcranial two-photon microscopy, we found that in both sensory and motor cortices, the elimination rates of dendritic spines were comparable between young (3–5 months) and mature adults (8–10 months), but seemed higher in old mice (>20 months), contributing to a reduction of total spine number in the old brain. During the process of motor learning, old mice compared to young mice had fewer new spines formed in the primary motor cortex. Motor training-evoked somatic calcium activity in layer 5 pyramidal neurons of the motor cortex was also lower in old than young mice, which was associated with the decline of motor learning ability during aging. Together, these results demonstrate the effects of aging on learning-dependent synapse remodeling and neuronal activity in the living cortex and suggest that synaptic deficits may contribute to age-related learning impairment.

## Introduction

The normal aging process is accompanied by a progressive decrease in cognitive and learning ability (Hedden and Gabrieli, 2004; Brayne, 2007; Deary et al., 2009). Neuropsychological tests have revealed that people older than 60 years of age often show impairments in certain types of memory, especially recall of recent events (Shimamura, 1994; Zelinski and Burnight, 1997). Elder people also experience increased difficulties in a variety of mental tasks (Rhodes, 2004). Aging-related cognitive decline leads to decreased quality of life and lowered ability to function. As the average lifespan is increasing worldwide, it becomes increasingly important to understand the neural mechanisms of age-related cognitive impairment.

Despite many efforts, the cellular basis of age-related cognitive decline remains unclear. It was once believed that a generalized neuronal loss in the cerebral cortex and deterioration of dendritic branching occurs during normal aging and contributes to cognitive impairment (Brody, 1955; Coleman and Flood, 1987). Yet stereological studies have documented minimal aging-related neuronal loss in the cortex and hippocampus (West et al., 1994; Morrison and Hof, 1997). It is now generally accepted that age-related cognitive decline is accompanied by minimum loss of neurons and subtle changes of neuronal arborization (Buell and Coleman, 1981; Flood et al., 1987; Flood, 1993; Morrison and Hof, 2002). On the other hand, changes in synapse number and plasticity have been reported in the brains of aging people and experimental animals (Terry and Katzman, 2001; Hof and Morrison, 2004; Burke and Barnes, 2006; Dickstein et al., 2007). Because neuronal circuit function is highly dependent on synaptic connectivity, age-related synapse loss and dysfunction likely contribute to the impairment in cognitive and learning capacities in the aged brain.

Dendritic spines are small protrusions extending from the shafts of dendrites, which represent postsynaptic sites of most excitatory synapses in the mammalian brain (Bhatt et al., 2009). During aging, an approximate 20–40% loss of dendritic spines has been demonstrated in the cortex and hippocampus of human and experimental animals (Mervis, 1978; Jacobs et al., 1997; Hedden and Gabrieli, 2004; Dumitriu et al., 2010). Consistent with the loss of dendritic spines, electrophysiological studies have shown that the field excitatory postsynaptic potential is reduced in aged rats (Barnes, 1979; Barnes and McNaughton, 1980; Luebke et al., 2004). Recent *in vivo* imaging studies reported that aging alters the dynamics of dendritic spines in the cortex of old mice (Mostany et al., 2013; Davidson et al., 2020). But how aging affects dendritic spine plasticity associated with learning remains unknown.

Previous studies have shown that novel sensory and learning experiences lead to rapid dendritic spine formation and elimination in functionally relevant cortical regions (Hofer et al., 2009; Xu et al., 2009; Yang et al., 2009; Kim and Nabekura, 2011; Lai et al., 2012; Hayashi-Takagi et al., 2015), raising the possibility that age-related decline in cognitive and memory function might attribute to the impairment in learning-dependent dendritic spine plasticity. To test this hypothesis, we examined the dynamics of dendritic spines in the mouse cortex *in vivo*. Our results showed that old mice (>20 months) had higher rates of spine elimination compared to young (3–5 months) and mature adults (8–10 months). During the process of motor learning, training-induced spine formation and somatic calcium activity were significantly lower in the motor cortex of old mice than young adults. These findings suggest that the ability of neurons to maintain and regenerate synapses declines during aging, which may contribute to learning and memory impairment.

## Materials and Methods

### Experimental Animals

*Thy1*-YFP-H mice (Stock No.: 003782) were purchased from the Jackson Laboratory and used for dendritic spine imaging experiments. *Thy1*-GCaMP6slow mice (Founder line 1) were used for Ca^2+^ imaging experiments (Cichon et al., 2020). Mice were group-housed in animal facilities at New York University Medical Center and Columbia University Medical Center. All animal procedures were carried out following protocols approved by Institutional Animal Care and Use Committees (IACUC) at New York University and Columbia University as consistent with National Institutes of Health (NIH) Guidelines for the Care and Use of Laboratory Animals.

### *In vivo* Imaging of Dendritic Spines and Data Analysis

The surgical procedure for transcranial two-photon imaging has been described previously (Yang et al., 2010). While the animal was under deep anesthesia induced by 100 mg/kg ketamine and 15 mg/kg xylazine, the skull surface was exposed with a midline scalp incision. A small skull region (~0.2 mm in diameter) located over the primary somatosensory cortex or the primary motor cortex was identified based on stereotaxic coordinates. A custom-made, stainless steel plate was glued to the skull with a central opening over the identified brain region. To create a cranial window for imaging, the skull surface was immersed in artificial cerebrospinal fluid (ACSF), and a high-speed drill and a microsurgical blade were used to reduce the thickness of the skull to approximately 20 μm under a dissection microscope. After the skull thinning, the animal was placed under an Olympus two-photon microscope with the laser tuned to the optimal excitation wavelength for YFP (920 nm). Low laser power (20–30 mW at the sample) was used during imaging to minimize phototoxicity. Stacks of image planes within a depth of 100 μm from the pial surface was collected with a 60× water-immersion objective (1.1 N.A.) at a digital zoom of 1.0–3.0. The step size was 2 μm for the initial low magnification image (1.0× zoom) for relocation at later time points and 0.75 μm for all the other experiments (3.0× zoom). After imaging, the steel plate was gently detached from the skull, and the scalp was sutured with 6–0 silk. The animals were returned to their home cages until the next view.

Data analysis was performed with NIH ImageJ software as described previously (Huang et al., 2016). The same dendritic segments were identified from three-dimensional image stacks taken at both time points. The number and location of dendritic protrusions were identified in each view. Filopodia were identified as long, thin structures without enlarged heads, and the rest of the protrusions were classified as spines. Spines were considered the same between two views based on their spatial relationship to adjacent landmarks. Spines in the second view were considered different if they were more than 0.7 μm away from their expected positions based on the first view. The formation or elimination rates of spines were measured as the number of spines formed or eliminated divided by the number of spines existing in the first view.

### Rotarod Training

An EZRod system with a test chamber (44.5 × 14 × 51 cm dimensions, Accuscan Instruments, Columbus, OH, USA) was used in this study. Animals were placed on the motorized rod (30 mm in diameter) in the chamber. The rotation speed gradually increased from 0 to 60 rpm for 3 min. The time latency and rotation speed were recorded when the animal was unable to keep up with the increasing speed and fell. Rotarod training was performed in one 30-min session (20 trials) per day. The performance was measured as the average speed animals achieved during the training session.

### Treadmill Training

A treadmill task was introduced to provide mild motor training, as well as to perform *in vivo* Ca^2+^ imaging and motor training at the same time. A custom-built treadmill (46 × 15 × 10 cm dimensions) was used to allow head-fixed mice to move their limbs freely on a moving belt. Following the onset of a training trial, the belt speed was gradually increased from 0 to 4 cm/s within ~3 s and maintained at 4 cm/s through the rest of the trial. Mice walked on the moving belt and adjusted their gait patterns progressively. To obtain footprints, mouse paws were coated with ink and a sheet of white construction paper was placed on top of the belt during treadmill training. Gait analysis was performed manually offline. The animal’s gait pattern was classified as drag, wobble, sweep, and steady run as previously described (Cichon and Gan, 2015). Treadmill performance was presented as the percentage of time spent in a steady run, averaged over five trials.

### Immunohistochemistry

Mice were deeply anesthetized and perfused with a phosphate-buffered solution (PBS) and 4% paraformaldehyde (PFA). The brain was removed and post-fixed in 4% PFA for 3 days and rinsed three times with PBS. Following gradient dehydration in 10%, 20%, and 30% sucrose solution at 4°C, the brain (from AP −0.4 to −1.2 mm) was sectioned with a Leica vibratome (VT 1000 S) at 50 μm thickness. Sections were post-fixed in 4% PFA for 1 h and washed three times with PBS. Sections were permeabilized and blocked at room temperature in 0.2% Triton X-100 and 5% goat or donkey serum in PBS for 1.5 h and then incubated overnight at 4°C with primary antibodies: rabbit anti-Iba1 (Wako, 019-19741, 1:2,000); mouse anti-GFAP (Sigma–Aldrich, AMAB91033, 1:1,000); rabbit anti-GFP (Abcam, ab6556, 1:1,500) and chicken anti-MAP2 (Abcam, ab5392). The sections were then washed three times with PBS and incubated for 2 h at room temperature with secondary antibodies: goat anti-rabbit Alexa Fluor 488 (1:500), donkey anti-rabbit Alexa Fluor 555 (1:500), donkey anti-mouse Alexa Fluor 488 (1:500), and goat anti-chicken Alexa Fluor 647 (1:500). The sections were washed as before and then mounted in a mounting medium containing DAPI (VectorLabs, H-2000) for confocal imaging.

### Confocal Imaging and Quantification

Confocal images were taken with a 20× objective at 1,024 × 1,024 pixels (0.62249 μm/pixel) using a Nikon Ti laser scanning confocal system. The fluorophores of DAPI, Alexa Fluor 488, and Alexa Fluor 647 were excited by 405, 488, and 640 nm laser, respectively. A *z*-stack of images was obtained at 2 μm step size and projected at a maximal intensity to generate the final three-channel images, which were then analyzed in ImageJ. For *Thy1*-GCaMP6s Line 1 mice, the mean fluorescent intensity of individual Alexa Fluor 488-positive cells (*F*_Anti-GFP_) in layer 5 (L5) of the cortex was normalized by that of Alexa Fluor 647 (*F*_Anti-MAP2_), where the expression level of neuronal skeleton marker MAP2 was used as an internal reference to correct the potential variations introduced by experimental procedures. The ratio (*F*_Anti-GFP_/*F*_Anti-MAP2_) was used to represent the expression level of GCaMP6s in individual neurons. The ratio (*F*_Anti-GFP_/ *F*_Anti-MAP2_) in individual MAP2-positive cells in wild type (WT) mice was quantified as a negative control.

### *In vivo* Ca^2+^ Imaging

*In vivo* Ca^2+^ imaging was performed in *Thy1*-GCaMP6s mice under awake states. The surgical procedure for preparing mice for awake imaging has been described previously (Yang et al., 2013). In brief, a head holder composed of two metal bars was attached to the animal’s skull to reduce motion artifact during imaging. Following surgical anesthesia and a midline scalp incision, the region of the skull located over the primary motor cortex was identified and marked with ink. The head holder was attached to the skull surface first with cyanoacrylate-based glue (Loctite 495) and then with dental acrylic cement, leaving the marked skull region in between the two metal bars. After the cement was completely dry, a craniotomy was made over the motor cortex and covered with a glass coverslip custom to the size (1.0–1.5 mm diameter) of the bone removed. The coverslip was glued to the skull to stabilize the exposed brain. Before imaging, mice were given 1 day to recover from the surgical anesthesia and habituated several times (10 min each time) to minimize the potential stress of head restraining and imaging.

*In vivo* Ca^2+^ imaging was performed using an Olympus two-photon system equipped with a Ti:sapphire laser (Mai Tai DeepSee; Spectra-Physics) tuned to 920 nm. To examine the somatic activity of L5 pyramidal neurons, Ca^2+^ images were collected at the depth of 500–600 μm below the pial surface, at a frame size of 256 × 256 pixels and a rate of 2 Hz, using a 25× objective immersed in ACSF. Image acquisition was performed using Olympus FV1000 software and analyzed *post hoc* using NIH ImageJ software. Ca^2+^ analysis was performed as previously described (Zhao et al., 2018). Δ*F*/*F*_0_ was calculated as (*F − F*_0_)/*F*_0_, where *F*_0_ is the baseline fluorescence averaged over a 2-s period corresponding to the lowest recording signal over the resting period (i.e., treadmill off). The average integrated Ca^2+^ activity was the average of Δ*F*/*F*_0_ over 2.5 min recording.

### Statistics

Prism software (GraphPad 7.0) was used to conduct statistical analysis. Data were presented as mean ± SEM. For spine imaging and calcium imaging data, tests for differences between populations were performed using two-tailed Mann–Whitney’s test. For behavioral data, tests for differences between groups were performed using the two-tailed Student’s *t*-test. Immunostaining data were compared using Kolmogorov–Smirnov tests. Significant levels were set at *P* ≤ 0.05.

## Results

### More Spines Are Eliminated in the Sensory Cortex of Old Mice

**Figure 1:**
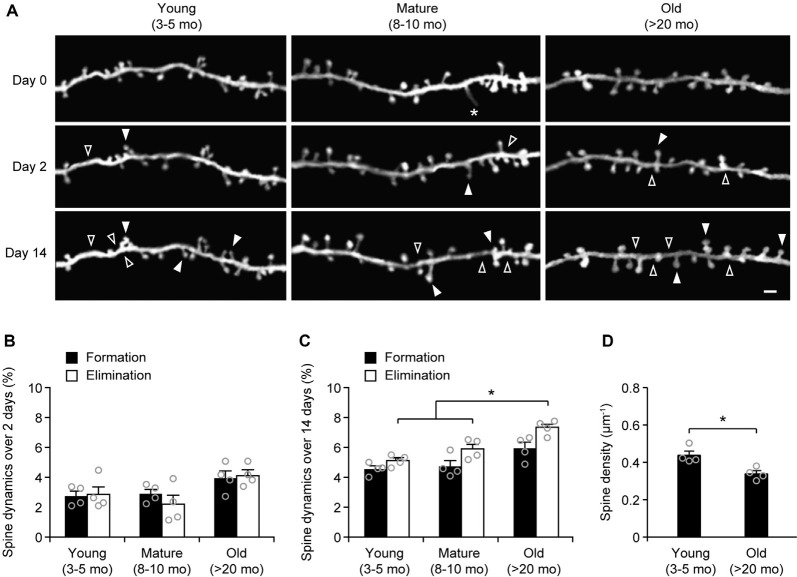
Aging increases dendritic spine elimination in the sensory cortex. **(A)** *In vivo* time-lapse imaging of the same dendritic segments over 2 and 14 days in the sensory cortex of young and old mice. Filled and empty arrowheads indicate dendritic spines that were formed and eliminated relative to the first view, respectively. Asterisks indicate filopodia. Scale bar, 2 μm. **(B)** Percentages of dendritic spines that were formed and eliminated over 2 days. **(C)** Percentages of dendritic spines that were formed and eliminated over 14 days. **(D)** The density of dendritic spines on the apical tuft dendrites of L5 pyramidal neurons in young adult and old mice. Throughout, individual circles represent data from a single mouse. Summary data are presented as mean ± SEM. *P < 0.05 by two-tailed Mann–Whitney test.

To examine age-related changes in dendritic spine plasticity, we first compared spine turnover rates in the cortex of young adult (3–5 months of age), mature adult (8–10 months of age), and old mice (>20 months of age). Apical dendrites of L5 pyramidal neurons in the sensory cortex of *Thy1*-YFP-H transgenic mice were repeatedly imaged using transcranial two-photon microscopy (Figure 1A). Consistent with previous studies (Grutzendler et al., 2002; Yang et al., 2009), we found a small and equivalent degree of dendritic spine formation and elimination over 2 days in young, mature, and old brains (Figures 1A,B). In young adult mice, 2.7 ± 0.3% dendritic spines were formed and 2.9 ± 0.6% were eliminated over 2 days (658 spines, *n* = 4 mice). Similar turnover rates were found in mature adult mice over 2 days (formation: 2.9 ± 0.3%; elimination: 2.2 ± 0.6%; 569 spines, *n* = 4 mice). In old mice, the rates of spine formation (3.9 ± 0.5%) and elimination (4.11 ± 0.4%) increased slightly (515 spines, *n* = 4 mice), although not significantly different compared to young and mature adult mice.

To determine long-term changes of dendritic spines, we repeatedly imaged dendritic segments in the sensory cortex over a 2-week interval (Figure 1C). We found that 4.6 ± 0.2% of dendritic spines were formed and 5.1 ± 0.2% were eliminated over 2 weeks in young adult mice (639 spines, *n* = 4 mice). In mature adults, spine formation and elimination over 2 weeks were 4.7 ± 0.4% and 5.9 ± 0.3%, respectively, compared to those in young adults (Figure 1C). Notably, in old mice, the percentage of spines eliminated (7.4 ± 0.3%) over 2 weeks was significantly higher than those in young and mature adults. Moreover, the percentage of spines eliminated over 2 weeks was slightly, but not significantly, higher than the percentage of spines formed (7.4 ± 0.3% vs. 5.9 ± 0.4%, *P* = 0.0571) in old mice, leading to a trend of spine loss in the sensory cortex. Consistently, dendritic spine density in layer 1 of the sensory cortex was significantly lower in old mice as compared with young adult mice (Figure 1D). Together, these results indicate a net loss of dendritic spines in the old sensory cortex due to a higher number of spines being eliminated than formed.

**Figure 2:**
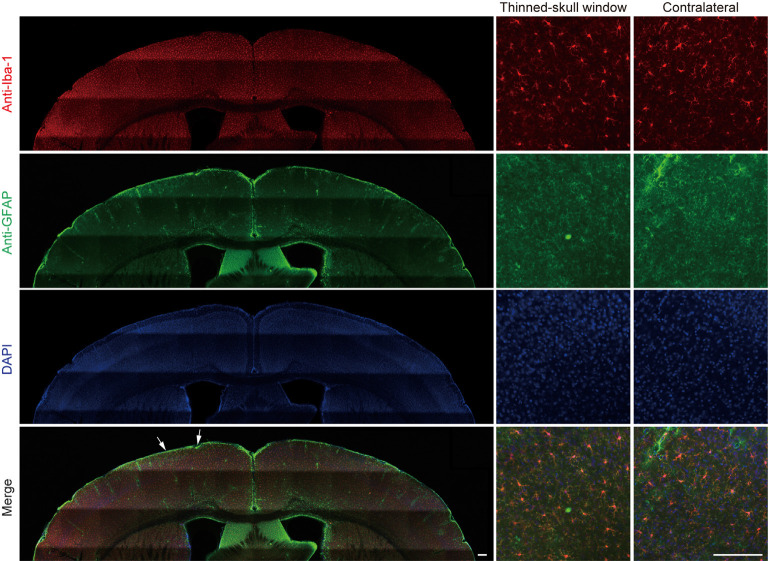
No obvious glial activation is observed under the thinned-skull window. Two days after thinning-skull surgery in 20-month-old mice, iba1-stained microglia appeared normal both on the contralateral control side and under the thinned-skull window. Little GFAP expression was found under the thinned-skull window (between arrows) or on the contralateral control side. Scale bar, 200 μm.

In a separate experiment, we stained microglia and astrocytes in the cortex of old mice after the thinning-skull surgery. Consistent with the previous report that the properly performed thinning-skull surgery does not cause glial activation (Xu et al., 2007), we did not observe noticeable changes in microglial morphology and density in the old cortex within 2 days after the surgery (Figure 2). Moreover, the expression levels of glial fibrillary acidic protein (GFAP) in the cortical areas under the cranial window was comparable to those in the contralateral hemisphere, indicating no activation of astrocytes (Figure 2). These results suggest that the increased turnover of spine dynamics in the old cortex is unlikely due to the surgery-induced glial activation.

### More Spines Are Eliminated in the Motor Cortex of Old Mice

**Figure 3:**
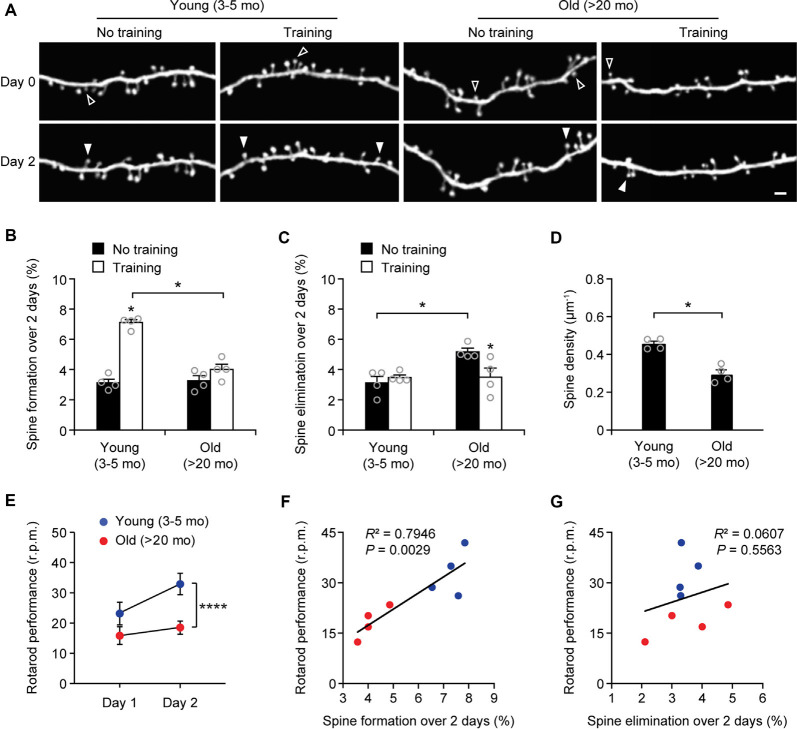
Aging decreases learning-induced spine formation in the motor cortex. **(A)** *In vivo* time-lapse imaging of the same dendritic segments over 2 days in the primary motor cortex of young and old mice, with or without motor training. Empty and filled arrowheads indicate individual spines that were eliminated or newly formed, respectively. Scale bar, 2 μm. **(B)** Percentages of dendritic spines formed over 2 days in young and old mice. **(C)** Percentages of dendritic spines eliminated over 2 days in young and old mice. **(D)** The density of dendritic spines on the apical tuft dendrites of L5 pyramidal neurons in young adult and old mice. **(E)** Rotarod performance in young and old mice (*n* = 8 mice per group). After 2-day training, old mice showed less performance improvement than young mice (Day 1: *t* = 2.065; *P* = 0.0580; Day 2: *t* = 6.230; *P* < 0.0001). **(F,G)** Following 2-day training, animals’ performance on the rotarod strongly correlated with the number of new spines formed but not with the number of spines eliminated (Pearson correlation). Throughout, individual circles represent data from a single mouse. Summary data are presented as mean ± SEM. *P < 0.05, ****P < 0.0001 by two-tailed Mann–Whitney test in panels **(B–D)** and two-tailed Student’s *t*-test in panel **(E)**.

Next, we measured the rates of spine turnover in the primary motor cortex during aging. Previous studies have shown that the rates of spine turnover are comparable across different cortical regions in adult mice (Zuo et al., 2005). Consistent with those studies, we found that 3.1 ± 0.2% and 3.1 ± 0.4% of dendritic spines were formed and eliminated, respectively, over 2 days in the motor cortex of young adult mice (539 spines, *n* = 4 mice; Figures 3A–C), comparable to those in the sensory cortex at the same age (Figure 1B). There were no differences in the rates of spine formation in the motor cortex between young adults and old mice (3.1 ± 0.2% vs. 3.3 ± 0.3%, *P* = 0.9714). In the motor cortex of old mice, the rate of spine elimination increased slightly as compared to that in young adults (5.2 ± 0.2% vs. 3.1 ± 0.4%, *P* = 0.0286) and was significantly higher than the rate of spine formation in the same mice (*P* = 0.0286; Figures 3A–C). In line with the trend of more spines being eliminated than formed in the old motor cortex, dendritic spine density in layer 1 of the motor cortex was significantly lower in old mice as compared with young adult mice (Figure 3D). These results indicate that during aging, a loss of dendritic spines also occurs in the motor cortex.

### Motor Learning-Induced Spine Formation Is Reduced in Old Mice

Experience-dependent remodeling of synaptic connections is essential for learning and memory. Previous studies have shown that motor skill learning promotes the rapid formation of new spines in the motor cortex and the extent of spine remodeling strongly correlates with behavioral improvement after learning (Yang et al., 2009; Liston et al., 2013). To examine the effects of aging on motor learning-dependent spine plasticity, we trained young adult and old mice on the rotarod task for 2 days. Dendritic spines in the primary motor cortex were imaged before and after rotarod training to determine learning-induced spine formation and elimination (Figure 3). Consistent with previous findings (Yang et al., 2009, 2014), we found that in 4-month-old adult mice, rotarod training over 2 days significantly increased the formation of new spines in the motor cortex (7.1 ± 0.2% vs. 3.1 ± 0.2%; 605 spines, 4 mice; *P* = 0.0286; Figures 3A,B). By contrast, motor training had little effect on spine formation in old mice (4.0 ± 0.4% vs. 3.3 ± 0.3%; *P* = 0.0857). The degree of learning-induced spine formation was significantly lower in old mice as compared to that in young adults (*P* = 0.0286), indicating that learning-induced spine formation is compromised during aging. Moreover, we found that motor training for 2 days had no significant effect on dendritic spine elimination in young adult mice (3.5 ± 0.2% vs. 3.1 ± 0.4%; *P* = 0.6286) but lowered the elevated spine elimination in old mice (3.5 ± 0.6% vs. 5.2 ± 0.2%; *P* = 0.0286; Figures 3A,C), suggesting a beneficial effect of motor training in preventing age-related synapse loss.

Consistent with the reduction of new spine formation after learning, we found that old mice compared to young adults showed less performance improvement after training in the rotarod task (Figure 3E). Notably, we observed a strong correlation between the percentages of new spines formed over 2-day training and the animals’ performance on the rotarod on day 2 (Pearson correlation: *R*^2^ = 0.7946, *P* = 0.0029; Figure 3F). There was no significant correlation between the rates of spine elimination over 2 days and the animals’ rotarod performance on day 2 (Pearson correlation: *R*^2^ = 0.0607, *P* = 0.5563; Figure 3G).

### Motor Training-Evoked Neuronal Activity Is Lower in the Cortex of Old Mice

**Figure 4:**
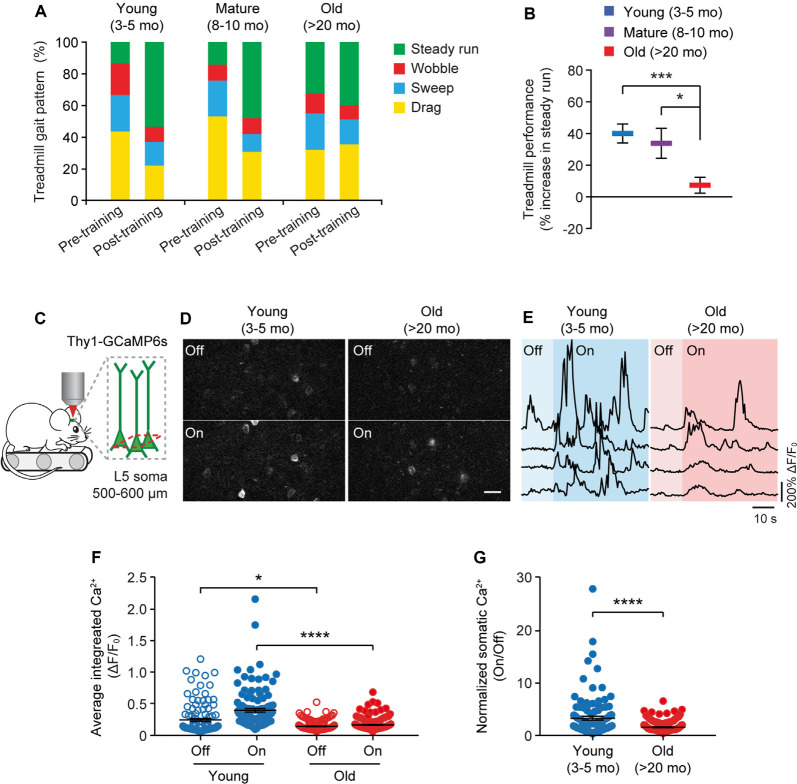
Aging decreases neuronal calcium activity in the motor cortex during resting and movement states. **(A)** Analysis of the animals’ gait patterns when walking on a treadmill. Old mice displayed higher proportions of drag, wobble, and sweep but less steady run after 1-h training. **(B)** Treadmill performance in young adults, mature adults, and old mice. Treadmill performance is expressed as a percent increase in the fraction of time spent in a steady run between post-training and pre-training (*n* = 10, 8, 13 mice per group). **(C)** Schematic showing two-photon Ca^2+^ imaging in the primary motor cortex of head-restrained mice walking on a treadmill. **(D)** Representative images of L5 somas expressing GCaMP6s when the treadmill is off and on. Scale bar, 20 μm. **(E)** Calcium fluorescence traces of representative pyramidal somas in young adult and old mice. **(F)** The average integrated activity of somatic calcium transients while the treadmill is off and on (Young: *n* = 116 cells from 6 mice; Old: *n* = 187 cells from 9 mice). **(G)** Normalized somatic calcium activity in young adult and old mice during treadmill training. Individual circles represent data from a single cell. Summary data are presented as mean ± SEM. *P < 0.05, ***P < 0.001, ****P < 0.0001 by two-tailed Student’s *t*-test in panel **(B)** and two-tailed Mann–Whitney tests in panels **(F,G)**.

Because the neuronal activity is critical for regulating dendritic spine plasticity, we next examined the impact of aging on the activity of cortical neurons during behavior. In this experiment, head-restrained mice were trained on the treadmill task on the stage of a two-photon microscope (Figure 4). With the belt moving at a walking speed, the treadmill task is mild in intensity and requires little physical strength to achieve. Consistent with previous studies (Huang et al., 2016), naive mice displayed large percentages of untrained gait feature, such as drag, wobble, sweep, when they first walked on the treadmill, whereas the proportion of trained feature (i.e., steady run) increased after training (Figure 4A). Five hours after initial 1-h training, both young and adult mice showed substantial increases in the steady run in their gait patterns (post-training vs. pre-training: young, *P* < 0.0001; mature, *P* = 0.009, paired *t*-test), whereas old mice showed less improvement in treadmill performance (Figure 4B).

Using *in vivo* two-photon imaging, we recorded the Ca^2+^ activity of L5 pyramidal neurons in the primary motor cortex using transgenic mice (*Thy1*-GCaMP6s line 1) expressing the genetically encoded Ca^2+^ indicator GCaMP6s in L5 pyramidal neurons of the cortex (Figure 4C). We found that under the quiet resting state (i.e., treadmill off), the average integrated Ca^2+^ activity (Δ*F*/*F*_0_) of L5 somas was significantly lower in old mice compared to young adults (0.13 ± 0.01 vs. 0.24 ± 0.02; *P* = 0.0485; Figures 4D–F). When mice were trained on the treadmill (i.e., treadmill on), there was a marked increase in somatic Ca^2+^ levels relative to resting states in young adult mice (0.39 ± 0.03 vs. 0.24 ± 0.02; *P* < 0.0001; Figures 4D–G). Compared to young adults, both the absolute (0.15 ± 0.01 vs. 0.39 ± 0.03; *P* < 0.0001) and normalized (on/off: 1.41 ± 0.07 vs. 3.15 ± 0.36; *P* < 0.0001) levels of somatic Ca^2+^ during treadmill training were substantially lower in old mice (Figures 4F,G).

**Figure 5:**
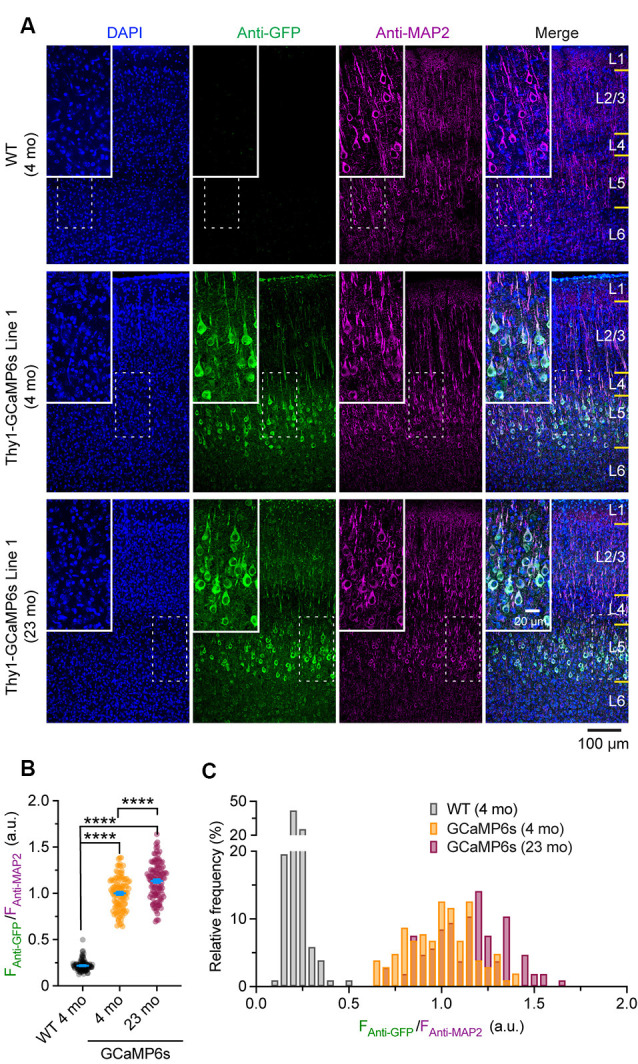
Characterization of GCaMP6 expression in young adult and old mice. **(A)** Representative coronal sections of the mouse cortex stained for GFP and MAP2 in 4-month-old wild type (WT), 4- and 23-month-old transgenic *Thy1*-GCaMP6s line 1 mice. **(B)** Expression levels of GCaMP6s (*F*_Anti-GFP_/*F*_Anti-MAP2_) in individual L5 pyramidal neurons. **(C)** Distribution of GCaMP6s levels in individual neurons. Summary data are presented as mean ± SEM. ****P < 0.0001 by Kolmogorov–Smirnov tests.

To rule out the possibility that the reduction of Δ*F*/*F*_0_ in old mice might be due to extensive changes of cytoplasmic GCaMP6s concentration, we performed immunostaining to characterize the protein levels of GCaMP6s in individual cells. As shown in Figure 5A, at both 4 and 23 months of age, GCaMP6s was primarily expressed in L5 of the motor cortex in *Thy1*-GCaMP6s line 1 mice. We found that the expression pattern of GCaMP6s was largely similar between young adults and old mice (Figures 5A–C). The average level of GCaMP6s per cell in old mice was about 1.1-folds of that in young adults (Figures 5A–C). Thus, the cellular concentration of GCaMP6s changed little between young adult and old mice, allowing for an appropriate comparison of Δ*F*/*F*_0_ between them. Together, these results show that aging decreases pyramidal neuronal activity in the motor cortex under both resting and movement states.

## Discussion

Aging decreases synapse number and function in the brain and drives a progressive decline in cognition, yet the link between synaptic alterations and learning impairment during normal aging remains unclear. Using the *in vivo* imaging approach, we examined dendritic spine formation and elimination in response to motor skill learning in young adult and old mice. We found that spine elimination was higher in old than young adult mice under basal conditions, while motor learning-induced spine formation was reduced in old mice. Additionally, we found that motor training-evoked neuronal activity in the motor cortex was markedly decreased in old mice. These findings reveal age-related alterations in synaptic plasticity and neuronal activity in the mouse cortex *in vivo*.

With the high-resolution two-photon imaging technique and the behavioral learning assays, we sought to answer several long-standing questions regarding the effects of aging on synaptic plasticity in the mammalian brain. Specifically, how does aging affect the degree of synapse formation and elimination in the living cerebral cortex? Does advanced age compromise synaptic remodeling associated with learning and memory formation? To address these questions, we first compared the dynamics of dendritic spines between young adults (3–5 months), mature adults (8–10 months), and old mice (>20 months) in the basal state (i.e., no training). We found that in the sensory cortex, the elimination rate of existing spines is comparable between young and mature adults, but significantly higher in old mice. Aging has no significant effect on the rate of spine formation under basal conditions. A similar finding was also observed in the motor cortex. These findings support the notion that synapse loss is a prominent feature of the aged brain and suggest that the disruption of the synaptic connectivity established early in life may contribute to the decline of cognitive function during aging. It is worth noting that the results of the present study seem to contradict a recent report of elevated dendritic spine density and dynamics in the primary motor cortex of aged mice (Davidson et al., 2020). Such discrepancy can be attributed to different transgenic mouse lines (*Thy1*-YFP-H vs. *Thy1*-GFP line M) and types of observation windows (thinned skull vs. open skull) used for spine imaging, as well as different data analysis methods (manual vs. automated). Indeed, previous studies have linked different observation windows to different turnover rates of dendritic spines (Xu et al., 2007).

It is well established that sensory and behavioral experiences have a profound impact on synaptic connections in cortical circuits (Wiesel, 1982; Knott et al., 2002; Xu et al., 2009; Lai et al., 2012; Yang et al., 2014; Ma et al., 2016). We have previously shown that rotarod skill learning induces the rapid formation of new spines over days as well as the elimination of existing spines over a longer period (Yang et al., 2009). Importantly, the extent of spine remodeling correlates with behavioral improvement after learning, underscoring a critical role of structural synaptic plasticity in learning and memory formation. In this study, we examined dendritic spine formation and elimination in response to motor skill learning in the motor cortex of young adult and old mice. We found that in young adults, motor-learning induced a ~5% increase in new spine formation. By contrast, old mice did not show an obvious increase in spine formation over the 2 days of motor training. This decrease of learning-induced new spine formation correlated with the compromised motor learning abilities in old mice. These results suggest that the reduction of learning-dependent synaptic plasticity may play a role in age-related cognitive decline in old mice, although the weakening of other organs, such as muscle and sensory organs, cannot be excluded.

Another interesting finding from the current study is that motor training improves the stability of existing spines in the old motor cortex, supporting the beneficial effects of exercise on brain function. It is well acknowledged that exercise counteracts cognitive decline in aging and neurodegenerative diseases (Ahlskog et al., 2011). Exercise has been shown to enhance learning and hippocampal neurogenesis in aged mice (van Praag et al., 2005). At the level of synapses, exercise attenuates age-related changes in neuromuscular synapses (Valdez et al., 2010; Nishimune et al., 2012) and hippocampal synapses (Siette et al., 2013) in rodents. In conformity with these studies, our results provide evidence that motor training may help maintain synaptic connectivity in the old brain.

By performing Ca^2+^ imaging in the motor cortex of mice performing a treadmill task, we found that motor training-evoked neuronal activity is markedly lower in old mice relative to young adults. Together with the finding of decreased dendritic spine formation after motor training, these results suggest that cortical circuits in the old brain may be less responsive to modulation by learning experiences, and the plastic reserve of the brain declines during aging. Many age-related molecular changes in the brain and blood may contribute to the reduction of neuronal activity and synaptic plasticity in the cortex. Previous studies have shown that the amounts of synaptic proteins such as synaptophysin (Smith et al., 2000) and NMDA receptor subunits (GluN1, GluN2B; Magnusson et al., 2002) are decreased during aging. Moreover, age-related decline in protein expression of GluN1 and GluN2B subunits in both the frontal cortex and hippocampus correlates with impaired memory function in old rodents (Magnusson et al., 2007; Zhao et al., 2009). Additionally, emerging studies suggest that young blood contains specific factors that mediate age-associated changes in brain function (Katsimpardi et al., 2014; Middeldorp et al., 2016). A recent study reported that systemic factors enriched in young serum, such as thrombospondin-4 and SPARCL1, can act directly on neurons to promote synapse formation and NMDA receptor recruitment in cultured cells (Gan and Südhof, 2019). It would be interesting to examine whether the administration of these systemic factors into the old brain could improve synaptic plasticity and function *in vivo*.

It is important to point out that all the imaging experiments in this study were performed using fluorescence-expressing transgenic animals (*Thy1*-YFP-H and *Thy1*-GCaMP6s). Although we expect that these experiments will provide important information on age-related alterations of dendritic spine plasticity and neuronal function, some of the results could be confounded by the long-term expression of fluorescent protein in neurons. Future studies using other labeling methods, such as virus-mediated fluorescence expression, may help address these concerns.

In summary, we found that in the cortex of old mice, more dendritic spines were eliminated under basal conditions and fewer new spines were generated after learning. These findings reveal the effects of aging on the formation and maintenance of synaptic connections and provide the missing links between synaptic alterations and learning impairment during normal aging.

## Data Availability Statement

The original contributions presented in the study are included in the article, further inquiries can be directed to the corresponding author/s.

## Ethics Statement

The animal study was reviewed and approved by Institutional Animal Care and Use Committees (IACUC) at New York University Medical Center and Columbia University Medical Center.

## Author Contributions

LH and GY designed research studies and wrote the manuscript. LH, HZ, KC, and GY conducted experiments. LH, HZ, KC, XC, and GY analyzed data. All authors contributed to data interpretation. All authors contributed to the article and approved the submitted version.

## Conflict of Interest

The authors declare that the research was conducted in the absence of any commercial or financial relationships that could be construed as a potential conflict of interest.
